# Retraction: Predictive modeling of surgical outcomes in lumbar stenosis and degenerative scoliosis using 3D gait-based spine-pelvic compensation analysis

**DOI:** 10.3389/fsurg.2026.1867369

**Published:** 2026-05-07

**Authors:** Frontiers Editorial Office

The journal retracts the 14 July 2025 article cited above.

Following publication, concerns were raised regarding the validity of the data in the article. The authors failed to provide a satisfactory explanation during the investigation, which was conducted in accordance with Frontiers' policies.

This retraction was approved by the Chief Editors of Frontiers in Surgery and the Chief Executive Editor of Frontiers. The authors received a communication regarding the retraction and had a chance to respond. This communication has been recorded by the publisher.

